# A State-Dependent Elasto-Plastic Model for Hydrate-Bearing Cemented Sand Considering Damage and Cementation Effects

**DOI:** 10.3390/ma17050972

**Published:** 2024-02-20

**Authors:** Huidong Tong, Youliang Chen, Xi Du, Siyu Chen, Yungui Pan, Suran Wang, Bin Peng, Rafig Azzam, Tomas Manuel Fernandez-Steeger

**Affiliations:** 1 Department of Civil Engineering, School of Environment and Architecture, University of Shanghai for Science and Technology, Shanghai 200093, China; 2 Department of Engineering Geology and Hydrogeology, RWTH Aachen University, 52064 Aachen, Germany; 3 School of Cyber Science and Technology, Shandong University, 72 Binhai Road, Jimo District, Qingdao 266237, China; 4 Department of Underground Architecture and Engineering, Tongji University, Shanghai 200093, China; 5 Institut für Angewandte Geowissenschaften, Technische Universität Berlin, 10587 Berlin, Germany

**Keywords:** hydrate-bearing cemented sand, state-dependent, critical state model, elastic-plastic, damage and cementation

## Abstract

In order to optimize the efficiency and safety of gas hydrate extraction, it is essential to develop a credible constitutive model for sands containing hydrates. A model incorporating both cementation and damage was constructed to describe the behavior of hydrate-bearing cemented sand. This model is based on the critical state theory and builds upon previous studies. The damage factor *D*_s_ is incorporated to consider soil degradation and the reduction in hydrate cementation, as described by plastic shear strain. A computer program was developed to simulate the mechanisms of cementation and damage evolution, as well as the stress-strain curves of hydrate-bearing cemented sand. The results indicate that the model replicates the mechanical behavior of soil cementation and soil deterioration caused by impairment well. By comparing the theoretical curves with the experimental data, the compliance of the model was calculated to be more than 90 percent. The new state-dependent elasto-plastic constitutive model based on cementation and damage of hydrate-bearing cemented sand could provide vital guidance for the construction of deep-buried tunnels, extraction of hydrocarbon compounds, and development of resources.

## 1. Introduction

Natural gas hydrates are seen as a novel and ideal energy source with environmental value in the twenty-first century because of their wide distribution, vast geologic reserves, high energy density, and minimal environmental pollution [1,2]. Natural gas hydrates are crystals of water and natural gas, primarily methane, and the detected reserves are widely distributed [3,4] in marine sediments and permafrost on Qingdao Plateau along the Sichuan-Tibet Railway [5]. The wetland structure in the gas hydrate-rich region of the Tibetan Plateau has also been anthropogenically damaged by the gas hydrate exploration activities that have recently taken place in China’s onshore area [6,7,8,9]. The microstructure and elastic properties of the rocks [10,11,12,13,14] in sedimentary strata are altered by the presence of gas hydrates. Natural gas hydrates decompose during extraction, weakening the mechanical qualities of the strata that contain them. This causes deformation and destroys the reservoirs, which in turn causes major geological disasters like landslides, stratigraphic deformation, and collapse.

The effect of hydrates on the mechanical properties of soils is primarily based on both filling and cementation, which results in energy soils exhibiting properties similar to those of dense sandy or cemented soils [15,16,17]. According to research, hydrates are primarily distributed in four ways in natural energy soils: pore filling, cementation, wrapping, and skeletal support [18,19,20]. According to Brown et al. [21], the breakdown of hydrates weakens the cementation of soil particles, increases porosity, and results in underconsolidated or loose sand soil. The development of a plausible constitutive model of hydrate-bearing cemented sand is of great value and significance for optimizing the efficiency and safety of natural gas hydrate exploitation.

To study the mechanical properties of hydrate-containing cemented sand, numerous researchers have produced hydrate-containing soils indoors [22,23,24,25,26]. By putting methane gas into sand with varying water contents and applying temperature and pressure conditions, the effects of various saturations, confining pressures and temperatures on the shear strength of energy soils were examined [27]. The gas saturation method was used to produce test specimens. As gas hydrate dissociates, the shear strength gradually decreases from 30% to 90% of the initial level [28], the shear zone thickens and the soil displays a brittle damage pattern [17]. Soil with more dispersed distribution of hydrates yields later, has higher peak strengths and slower development of cementation damage [29]. 

Experts and academics both domestically and internationally have conducted a number of studies on the constitutive model of hydrate-containing cemented soils, which may be broadly divided into three groups based on the experimental results: The nonlinear elastic constitutive model is the first kind. Using the least-squares method, Miyazaki et al. [22] determined the tangential Poisson’s ratio related to the matrix material and defined the tangential modulus expression related to hydrate saturation (*S*_h_). The effective circumferential pressure and hydrate saturation were taken into account when establishing the nonlinear elasticity principle model. In order to improve the model’s ability to represent the stress-strain relationship of naturally occurring soft clay soil, Wang et al. [30] introduced the concept of soil damage to modify the Duncan-Chang model. The second type is a damage constitutive model for hydrate-bearing cemented soils based on geotechnical damage theory [31,32,33,34]. The use of effective hydrate saturation was proposed to characterize the effect of hydrate storage mode [35,36]. Weibull distribution and residual strength correction coefficients were introduced into the damage statistical constitutive model [37]. Nevertheless, these models do not take into account the impact of gas hydrate cementation or alternative storage methods, and obtaining the Weibull parameters might be challenging. The third category is the elastic-plastic constitutive model. By considering the filling effect, cementation effect and other forms of fugacity of hydrates, the existing models such as the Cam-Clay model [24] and the uniform hardening model [38] are expanded accordingly. Uchida et al. [26] took into account the shear expansion and cohesion of the soil, introduced the concept of mechanical hydrate saturation related to the skeleton structure of the sediments, and transformed the yield surface equation of the modified Cam-Clay model to model the critical state of hydrate-bearing soils. Considering that hydrates have no effect on the residual strength of soils, parameters related to hydrate saturation were introduced to correct the hardening law [25]. Zhu et al. [39] introduced cementation stresses *s* in the uniform hardening model of structural soils under the basic framework of uniform hardening theory. Two parameters β (controlling the degree of strength of the cemented structure) and the parameter ω (controlling the degree of breakage of the cemented structure) related to the structural nature of the cementation were introduced into the elliptic-parabolic double yield surface model [40]. The influence of cementation was considered by translating the yield surface and the critical state surface based on the double hardening parameter model [41]. Li et al. connected the void ratio and the stress ratio to derive an equation of state-dependent dilatancy [42,43]. The critical state model of super-consolidated structural soils was revised in terms of an expression for the law of evolution of the structural strength *p* to represent the process of cemented structural damage of the soil [44]. 

A thorough knowledge of the damage and deformation features of sand affected by hydrates and weakened by hydrate cementation remains elusive after years of intensive research on hydrate-bearing cemented sand. The evolution law of the cementation stress gradual exertion process is not described by the majority of previously built models; instead, they explain the evolution law of the soil cementation stress decay process. Furthermore, the damage factor was not taken into account in the previously proposed hydrate-bearing cemented sand, making it impossible to adequately depict the deterioration of the cementation and the soil body damage. For hydrate-bearing cemented sand, we thus require a new state-dependent critical state model that takes cementation and damage into account.

An elastic-plastic model of hydrate-bearing cemented sand that takes cementation and damage into account was developed using the critical state theory, building on earlier research. To account for soil damage and the weakening of hydrate cementation, the damage factor *D*_s_, which is defined by plastic shear strain $\varepsilon_{d}^{p}$ is added. The damage is then incorporated into the cementation strength function *P*_h_ to describe the evolution law of the cementation stress progressive exertion process. By altering the shear dilatancy equations, yield surface equations, and hardening parameters of the constitutive model, a computer program was created to simulate the mechanisms of cementation and damage evolution as well as the stress-strain curves of hydrate-bearing cemented sand under various hydrate saturations *S*_h_, effective perimeter pressures CSP, and initial porosities *e*_0_. The results demonstrate the effectiveness of the model in reproducing the constitutive mechanical behavior of impaired soil cementation and soil deterioration with the same set of parameters when compared to the experimental data of earlier researchers. The conclusions can offer crucial directions for building deep-buried tunnel construction, hydrocarbon compound mining, and resource development.

## 2. Model Formulation

Soil was first treated as a work-hardening plastic material by Drucker et al. in 1957 [45]. Roscoe et al. found that the final portion of all loading paths lies on a unique surface and that the paths end with a unique critical porosity ratio line. The presentation of the Cam-Clay model and the modified Cam-Clay model marked the formalization of critical state theory [46,47]. And for the first time, strength theory and deformation theory (compression, dilatancy) were integrated into one framework. Roscoe et al. found that the deformation development of soil under external loading ends at a particular point regardless of the initial state and stress path. Soil in the large deformation stage of shear test tends toward the final critical condition, that is, the volume and total stress are unchanged, while the shear strain continues to develop and flow.

(1) $$\dot{\sigma }=0,{\dot{\varepsilon }}_{v}=0,{\dot{\varepsilon }}_{q}\neq 0$$

Since the solid particles are incompressible, the change in the volume of the soil specimens depends entirely on the change in the voids, i.e., the physical significance of the description of the soil specimens volume *v* and porosity *e* is equivalent. The effective stress in the critical state is constant, it is equivalent to the ratio of *q* and p* being constant.

(2) $$e=e_{c}={\hat{e}}_{c}(p)$$

(3) $$\eta ={\left(q/p^{*}\right)}_{c}=M_{c}$$

The drainage conditions and the state of the soil have no bearing on the requirements for attaining the critical state. Dimaggio and Sandler proposed the cap model based on Drucker et al.’s study and the Cambridge model [48,49]. A straightforward and useful method for easily obtaining the cap model coefficients from traditional geomechanical test data was presented by Huang and Chen (1990) [50].

When the soil is inside a yield surface, the model predicts an elastic behavior; after the yield surface is reached, a plastic behavior starts. The p*-q space is used for establishing the model. Deviator stress and mean effective stress are described as

(4) $$p*=\frac{\left({\sigma_{1}}^{*}+2{\sigma_{3}}^{*}\right)}{3}$$

(5) $$q=\sigma_{1}^{*}-\sigma_{3}^{*}$$

where ${\sigma_{1}}^{*}$ and ${\sigma_{3}}^{*}$ are the major and minor primary stresses.

For p* and q, the calculated conjugate strain rates are $d\varepsilon_{v}$ and $d\varepsilon_{d}$.

(6) $$d\varepsilon_{v}=d\varepsilon_{1}+2d\varepsilon_{2}$$

(7) $$d\varepsilon_{d}=\frac{2\left(d\varepsilon_{1}-d\varepsilon_{3}\right)}{3}$$

(8) $$d\varepsilon =d\varepsilon_{v}+d\varepsilon_{d}$$

(9) $$d\varepsilon_{d}=d\varepsilon_{d}^{e}+d\varepsilon_{d}^{p}$$

(10) $$d\varepsilon_{v}=d\varepsilon_{v}^{e}+d\varepsilon_{v}^{p}$$

where $d\varepsilon_{d}^{e}$ and $d\varepsilon_{d}^{p}$ are the elastic and plastic shear strain increments, and $d\varepsilon_{v}^{e}$ and $d\varepsilon_{v}^{p}$ are the elastic and plastic volumetric strain increments.

### 2.1. Implication of Damage

Mining leads to increased strain in soils, as well as performance degradation from hydrate cementation. Damage theory is applied to represent the degradation of hydrate cementation and the damage to cemented sand. It is hypothesized that the equation between the plastic shear strain $\varepsilon_{d}^{p}$ and the damage variable *D*_s_ of hydrate cementation and soils is as follows [51]:

(11) $$D_{s}=1-e^{s\varepsilon_{d}^{p}}$$

where *s* is the damage index, reflecting the damage rate of hydrate-bearing cemented sand.

### 2.2. Bonding and Debonding Regulations of Hydrate-Bearing Cemented Sand

The hydrates can increase the strength and cohesive strength of soils, giving them properties similar to structural and cemented sand [52]. In the critical state, the bonding is gradually weakened until it is completely destroyed, and only the friction strength plays a role [53,54].

The expression for the cementation factor *P*_h0_ is referenced [26]:

(12) $$p_{h0}=c{\left(S_{h}\right)}^{g}$$

Due to the very limited volumetric strain, the bonding damage *D*_s_ is introduced into the bonding strength function *P*_h_, which describes the evolution law of the gradual degradation of the bond stress [24]:

(13) $$\begin{array}{l} d\ln p_{h}=-v_{c}d\varepsilon_{d}^{p}\\ \ln p_{h}=-v_{c}d\varepsilon_{d}^{p}+C\\ p_{h}=C\cdot e^{-v_{c}d\varepsilon_{d}^{p}}\\ p_{h0}=c{\left(S_{h}\right)}^{g}\\ p_{h}=e^{-v_{c}d\varepsilon_{d}^{p}}\cdot c{\left(S_{h}\right)}^{g} \end{array}$$

where *v*_c_, *c* and *g* are model parameters. Introducing the damage factor *D*_s_ yields

(14) $${p^{*}}_{h}=p_{h}\cdot (1-D_{s}{)=e}^{-v_{c}d\varepsilon_{d}^{p}}\cdot c{\left(S_{h}\right)}^{g}\cdot (1-D_{s})$$

where ${p^{*}}_{h}$ is the bonding strength function considering damage. 

### 2.3. Elasticity Stage

The definition of the hydrate content in cemented sand is as listed below:

(15) $$S_{h}=\frac{V_{h}}{V_{h}+V_{0}}$$

where *V*_0_ and *V*_h_ stand for the pore space and hydrate volumes.

Based on the concept of elastic theory:

(16) $$d\varepsilon_{d}^{e}=\frac{dq}{3G}$$

(17) $$d\varepsilon_{v}^{e}=\frac{dp^{*}}{K}$$

(18) $$K=\frac{2(1+\mu )}{3(1-2\mu )}G$$

where *G* and *K* are the elastic shear and bulk parameters, μ is the Poisson’s ratio. Applying the hydrate-bearing cemented sand damage index *D*_s_ results in

(19) $$d\varepsilon_{vD_{s}}^{e}=\frac{dp^{*}}{K(1-D_{s})}$$

(20) $$d\varepsilon_{dD_{s}}^{e}=\frac{dq}{3G(1-D_{s})}$$

where $d\varepsilon_{vD_{s}}^{e}$, $d\varepsilon_{dD_{s}}^{e}$ are the increments of elastic volumetric and shear strain considering damage *D*_s_.

The shear stiffness resulting from the presence of hydrate and the sand skeleton’s shear modulus are added together to form *G*. It is presumed that the increase in shear stiffness $G_{h}\left(S_{h}\right)$ increases linearly with hydrate saturation [26].

(21) $$G_{h}\left(S_{h}\right)=G_{h}+\tau S_{h}$$

where G_h_(*S_h_*) is the shear stiffness index for hydrate-bearing cemented sand, *G_h_* is the shear stiffness index for sand particles, and τ is a model parameter.

**Figure 1:**
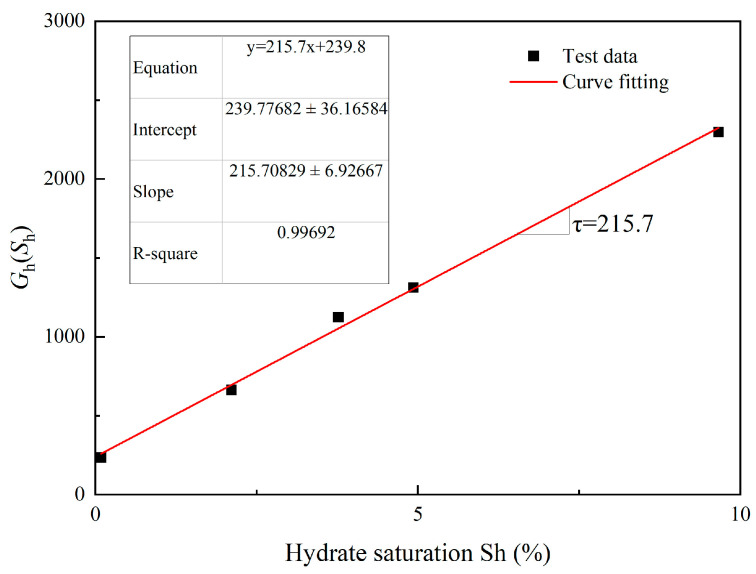
Best-fit curve for *G_h_*(*S_h_*) vs. hydrate saturation (source of experimental data: [55]).

In Figure 1, τ represents the gradient of the simulation line of experimental data from Clayton et al. [55]. Equation (21) illustrates the cumulative effect of the hydrate hole-filling and cementation processes on the hydrate-bearing cemented sand stiffness. It is impossible to ascertain the individual contributions of each mechanism to the rise in stiffness, though.

For sand particles, *G* can be represented using the empirical formula that follows [24]:

(22) $$G=\frac{G_{p}\cdot \sqrt{p^{*}p_{0}}\cdot {\left(2.97-e\right)}^{2}}{1+e}$$

where *P*_0_, *G*_p_ indicates the atmospheric pressure and shear stiffness parameter.

### 2.4. Yield Function Considering the Effects of Cementation and Damage

**Figure 2:**
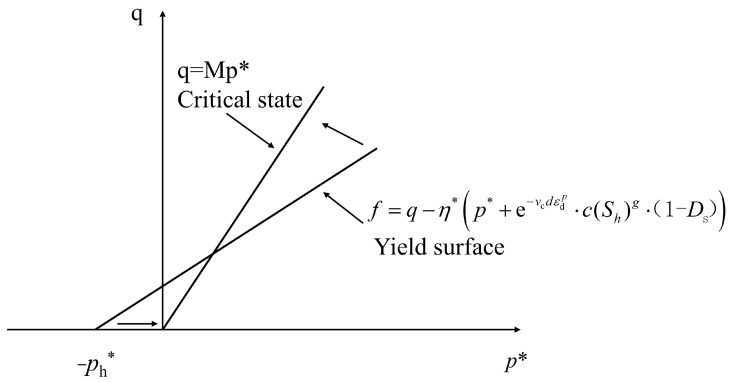
Yield surface and critical state line (after Shen et al., [24]).

In this paper, the cementation factor ${p^{*}}_{h}$ and damage factor *D*_S_ are introduced to characterize the weakening of hydrate cementation and sand damage. Prior to grain breaking, a steady stress-ratio loading only causes comparatively minor plastic volumetric strains [56]. The yield function for hydrate-bearing cemented sand could be expressed as

(23) $$f=q-\eta^{*}\left(p^{*}+e^{-v_{c}d\varepsilon_{d}^{p}}\cdot c{\left(S_{h}\right)}^{g}\cdot (1-D_{s})\right)$$

where $\eta^{*}$ is the stress ratio at yielding. Figure 2 shows the yield criterion for hydrate-bearing cemented sand in the p*-*q* space.

According to the theory of plasticity [57], the plastic consistency requirement, which is stated as follows, can be used to determine a loading index *L*

(24) $$L=\frac{1}{K_{p}}\left(\frac{\partial f}{\partial p^{*}}dp^{*}+\frac{\partial f}{\partial q}dq+\frac{\partial f}{\partial {p^{*}}_{h}}d{p_{h}}^{*}\right)=\frac{1}{K_{p}}\left(dq-\eta^{*}dp^{*}-\eta^{*}e^{-v_{c}d\varepsilon_{d}^{p}}\cdot c{\left(S_{h}\right)}^{g}\cdot (1-D_{s})\right)$$

where *K_p_* is the parameter of plastic hardening.

The $d\varepsilon_{v}^{p}$ and $d\varepsilon_{d}^{p}$ can then be expressed as [42]:

(25) $$d\varepsilon_{d}^{p}=L=\frac{1}{K_{p}}\left(dq-\eta^{*}dp^{*}-\eta^{*}e^{-v_{c}d\varepsilon_{d}^{p}}\cdot c{\left(S_{h}\right)}^{g}\cdot (1-D_{s})\right)$$

(26) $$d\varepsilon_{v}^{p}=LD=\frac{D}{K_{p}}\left(dq-\eta^{*}dp^{*}-\eta^{*}e^{-v_{c}d\varepsilon_{d}^{p}}\cdot c{\left(S_{h}\right)}^{g}\cdot (1-D_{s})\right)$$

where *D* is the dilatancy equation.

The damage factor *D_s_* is introduced and at the same time the effective stress is changed. Based on the assumption of equivalent strain, it can be obtained:

(27) $$d\varepsilon_{v}=d\varepsilon_{vD_{s}}^{e}+d\varepsilon_{vD_{s}}^{p}=\frac{dp^{*}}{K(1-D_{s})}+\frac{D}{K_{p}(1-D_{s})}\left(dq-\eta^{*}dp^{*}-\eta^{*}\cdot e^{-v_{c}d\varepsilon_{d}^{p}}\cdot c{\left(S_{h}\right)}^{g}\cdot (1-D_{s})\right)$$

(28) $$d\varepsilon_{q}=d\varepsilon_{dD_{s}}^{e}+d\varepsilon_{dD_{s}}^{p}=\frac{dq}{3G(1-D_{s})}+\frac{1}{K_{p}(1-D_{s})}\left(dq-\eta^{*}dp^{*}-\eta^{*}\cdot e^{-v_{c}d\varepsilon_{d}^{p}}\cdot c{\left(S_{h}\right)}^{g}\cdot (1-D_{s})\right)$$

### 2.5. Critical State

Hyodo et al. [58,59] examined the influence of density and hydrate saturation on the mechanical characteristics of the hydrate-bearing cemented sand. Additionally, it was presumable that each drained triaxial test ended with the approximate achievement of the critical state.

**Figure 3:**
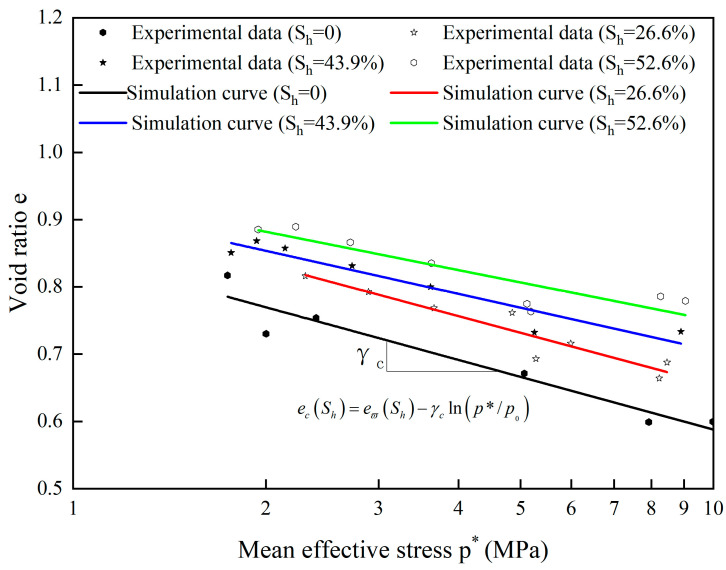
The lines of critical state for hydrate-bearing cemented sand (source of experimental data: Hyodo et al. [59]).

The influence of hydrate saturation on the critical state lines is depicted in Figure 3. It is evident that the average effective stress and the void ratio have a roughly linear relationship. As value of *S_h_* increases, the critical state lines moves upward, indicating that the hydrate-bearing cemented sand exhibits a tendency to dilate.

**Figure 4:**
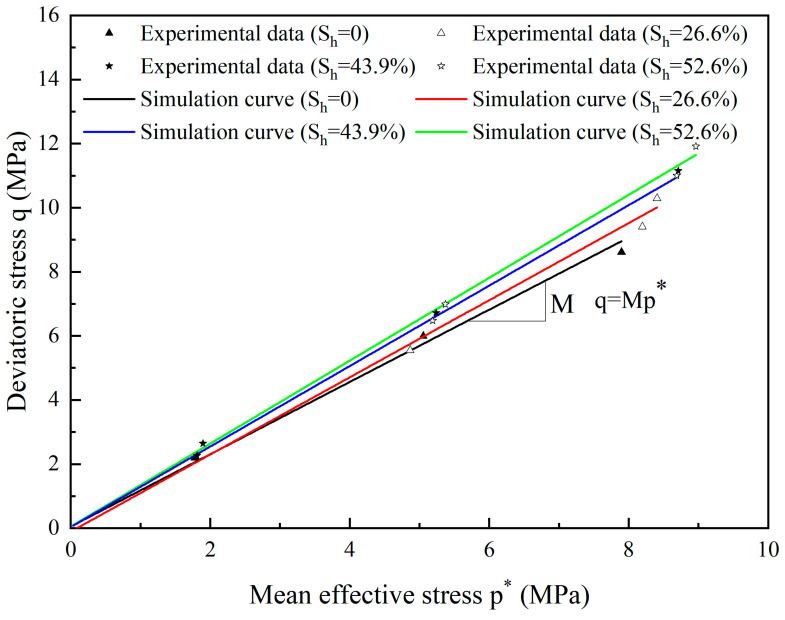
The lines of critical state for hydrate-bearing cemented sand (source of experimental data: Hyodo, Yoneda, et al. [59]).

The impact of *S*_h_ on the critical state lines is shown in Figure 4. The data from the experiment [59] matches well to a number of straight lines, the slope of which rises as one increases the value of *S*_h_.

**Figure 5:**
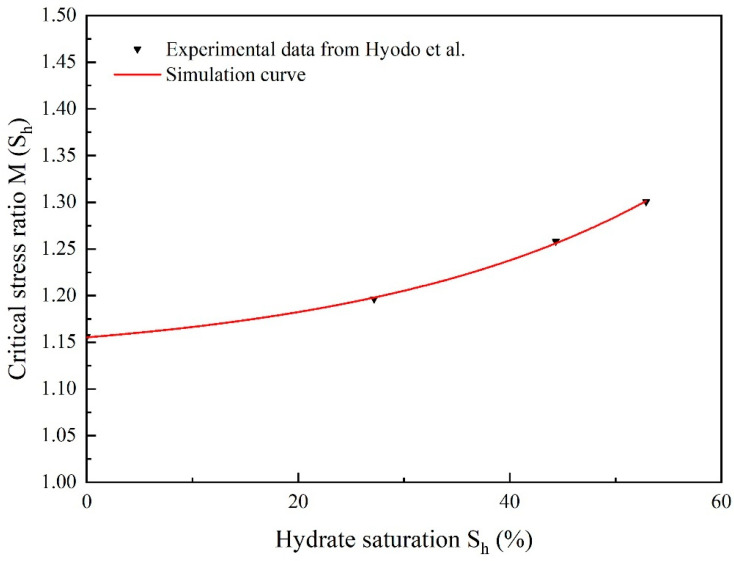
The fitted curve of the critical stress ratio of hydrate-bearing cemented sand with value of *S*_h_ (after Shen et al. [24]).

The change in stress ratios with hydrate saturation *M*(S_h_) at the critical state is shown in Figure 5. *M*(S_h_) rises in a nonlinear manner as hydrate saturation rises. Because there is more contact between the hydrate and the sand particles, more hydrate will add to sand-hydrate frictional resistance, the critical stress ratios increase more quickly as value of S_h_ approaches 60%.

It appears that the critical stress ratio *M*(S_h_) and critical state lines for hydrate-bearing cemented sand change with the hydrate saturation based on the earlier studies [24].

(29) $$e_{\varpi }\left(S_{h}\right)=e_{\varpi }+\varsigma S_{h}^{\kappa }$$

(30) $$e_{c}\left(S_{h}\right)=e_{\varpi }\left(S_{h}\right)-\gamma_{c}\ln\left(\frac{p^{*}}{p_{0}}\right)$$

(31) $$M\left(S_{h}\right)=M+\chi S_{h}^{\delta }$$

where *M* and *M*(S_h_) are the critical stress ratio for sand particles and the hydrate-bearing cemented sand, and $e_{\varpi }$, $\gamma_{c}$, ς, κ, χ and δ are model critical state parameters. 

### 2.6. State-Dependent Dilatancy Function

Tests have also shown that the dilatancy of the hydrate-bearing cemented sand is dependent upon bonding strength, hydration saturation, and stress ratio [60,61,62]. A general formulation for the dilatancy was put forward by Li and Dafalias [43].

(32) D=D(η, e, R, G)

where *R* is an internal state that is changeable beyond the void ratio, *G* stands for the intrinsic material constant, and η is the stress ratio.

There are two conditions that the generic dilatancy statement must fulfill. First of all, in a critical state, the dilatancy must be zero. Second, there is a chance to arrive at a “phase transformation state”, where *e* ≠ *e_c_*, η ≠ *M* and *D* = 0.

(33) $$D\left(R,G,e\neq e_{c},\eta \neq M\right)=0$$

(34) $$D\left(R,G,e=e_{c},\eta =M\right)=0$$

In order to account for the hydrate accumulation behavior in the dilatancy of sand, the following modified dilatancy expression is suggested (24):

(35) $$D=D\left(\eta^{*},e,{p^{*}}_{h},R,G\right)$$

where *p_h_* stand for the impacts on dilatancy of the cementing of hydrates.

Been and Jefferies [63] introduced a state factor $\varsigma =e-e_{c}$ to quantify the difference between the present situation and the critical state condition of sand particles by measuring the density. Li and Dafalias [43] introduced the state factor ς to express the state-dependent for the dilatancy equation.

(36) $$D=\frac{n_{0}}{M}\left(Me^{b\varsigma }-\eta \right)$$

where *b* and *n*_0_ are two model parameters.

In order to fulfill the conditions of Equations (33) and (34), the dilatancy equation is modified [24].

(37) $$D=\frac{n_{0}}{M\left(S_{h}\right)}\left[M\left(S_{h}\right)e^{b\varsigma \left(S_{h}\right)}n_{c}-\eta^{*}\right]$$

(38) $$\varsigma \left(S_{h}\right)=e-e_{\varpi }\left(S_{h}\right)$$

where $\varsigma \left(S_{h}\right)$ is the state index for the hydrate-bearing cemented sand. *n_c_* is an equation of the bonding strength ${p^{*}}_{h}$.

Considering the hydrate cementation weakening and soil damage as reflected in the bonding strength ${p^{*}}_{h}$. The damage factor *D*_s_ is introduced to propose the formulation for *n*_c_. *n*_c_ is employed to explain the cementing process on dilatancy.

(39) $$n_{c}=1-s\cdot D_{c}$$

### 2.7. Hardening Rule

A significantly revised constitutive equation is provided for the plastic index *K*_p_, as suggested by Li and Dafalias [43]. For a wide variety of confining pressures and void ratios, this kind of equation could effectively reflect the strain-hardening and strain-softening properties of hydrate-bearing cemented sand [43,64]:

(40) $$K_{p}=\frac{lGe^{j\varsigma \left(S_{h}\right)}}{\eta^{*}}\left[M\left(S_{h}\right)e^{-j\varsigma \left(S_{h}\right)}-\eta^{*}\right]$$

where *j* is a hardening rule index and *l* is a factor of the plasticity.

(41) $$l=l_{1}-l_{2}e_{0}$$

where *l*_1_ and *l*_2_ are model parameters. The plastic modulus is employing *l* to represent the impact of density.

## 3. Calibration of the State-Dependent Elasto-Plastic Model Parameters

There are nineteen parameters in the model. Ten of these are substrate sand mechanical properties parameters, which are listed in Table 1 and can be calibrated using the techniques suggested by Xiao et al. [54] and Li and Dafalias [43]. These parameters were derived by data fitting or formulae. Moreover, the calibrated values of these parameters are ratios, which are dimensionless, and are used to model and accurately predict the stress-strain relationships of hydrate-bearing cemented sand.

We thank the reviewer for this criticism. In Figure 1, τ represents the gradient of the simulation line of experimental data from Clayton et al. [55]. In Figure 3, $\gamma_{c}$ denotes the slopes of void ratio *e* and ln(p*/p_0_). The values can be found by fitting the experimental data [59]. The parameter $e_{\varpi }$ of Equation (30) could be calibrated by fitting the test data for the critical stress ratio and the critical state line in the *e*-p* plane [43]. In Figure 5, *M* denotes the critical state stress ratio, which can be obtained from the slope of the deviatoric stress *q* and mean effective stress p* curve fitted to the experimental data [59]. The parameter *n*_0_ could be calibrated based on the $\varepsilon_{v}-\varepsilon_{q}$ curves. Based on the experimental *q*-$\varepsilon_{q}$ curves, a suitable value of μ was firstly chosen and then the parameter *G*_p_ can be calculated. Once *G*_p_ has been determined, *l*, and therefore *l*_1_ and *l*_2_, could be calibrated on Equation (41) [43]. For hydrate-bearing cemented sand, nine more parameters are required (see Table 2); these are calibrated using the methodology described in the Shen et al. publication [24]. 

The damage index *s* represents the rate of damage to hydrate-bearing cemented sand. Since it is not possible to determine the damage index *s* directly through testing, this study examines the effects of various values of *s* on the simulation curves of hydrate-bearing cemented sand using the results of deviatoric stress q—axial strain $\varepsilon_{1}$ curves that were predicted with an efficient restriction pressure of 5 MPa and a hydrate saturation degree of *S*_h_ = 24.2%.

**Figure 6:**
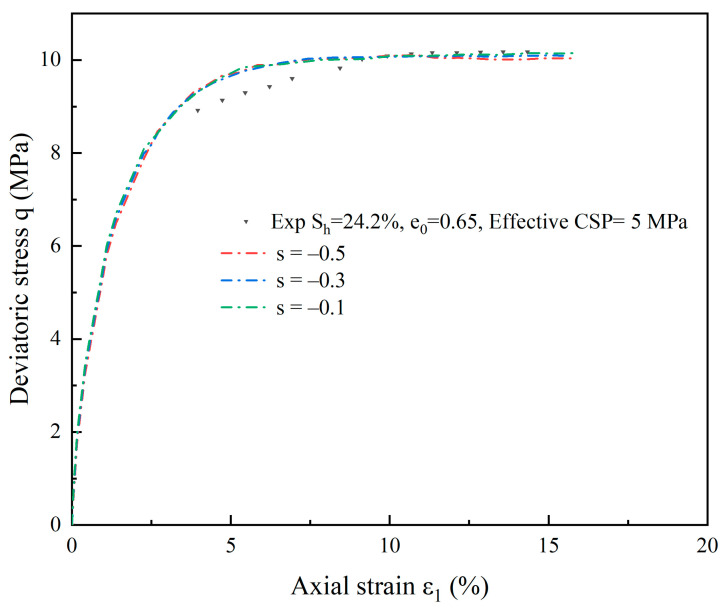
Effect of damage factor *s* on the deviatoric stress-axial strain curve (S_h_ = 24.2%, effective CSP = 5 MPa).

In the simulation process, the *s* value range is between 0 and −0.1. The *s* values are closer to the test results when 0.1. The *s* value can be adjusted according to the impact of different external factors on the rate of degradation of hydrates [51]. An analysis of Figure 6 demonstrates that the higher the value of *s*, the higher the peak intensity of the deviatoric stress-axial strain curve. The lower the value of s, the more evident the cementation’s weakening effect is, resulting in a smaller peak value overall. When the axial strain is small, the impact of the model parameter *s* on the q-$\varepsilon_{1}$ curve is small, and with an increase in axial strain, the impact on the q-$\varepsilon_{1}$ curve begins to appear.

## 4. Model Validation and Analysis

Utilizing the host material Toyoura sand for the drainage triaxial test, cylindrical frozen specimens with a diameter of 30 mm and a height of 50 mm were created by combining the volume of sand corresponding to the target density with the amount of water in the range of 0–60% saturation of the target hydrate [59]. Triaxial compression tests were conducted using initial porosities of around 40% and 45% at effective confining pressures of 1 MPa, 3 MPa, and 5 MPa and temperatures of 1 °C, 5 °C, and 10 °C at a shear rate of 0.1%/min.

**Figure 7:**
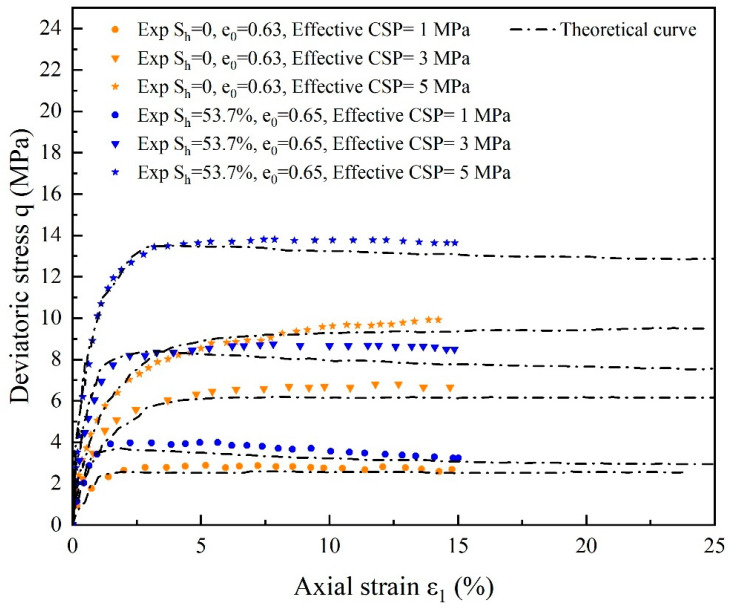
Comparison of experimental and theoretical curves of deviatoric stress for substrate sand with varying initial void ratios, hydrate saturation and effective restricting pressures (source of experimental data: Hyodo et al. [59]).

**Figure 8:**
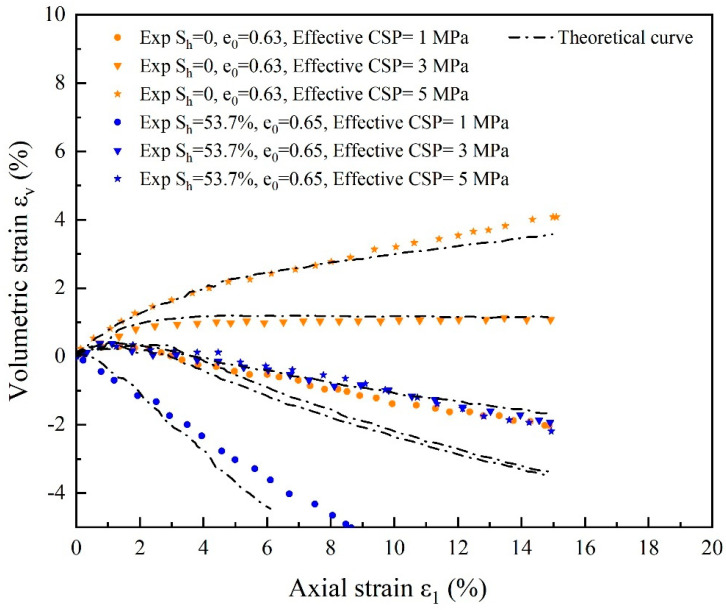
Comparison of experimental and theoretical curves of volumetric strain for substrate sand with varying initial void ratios, hydrate saturation and effective restricting pressures (source of experimental data: Hyodo et al. [59]).

The deviatoric stress and volumetric strain actual and theoretical curves for substrate sand and cemented sand with 53.7% hydrate saturation at various effective confining pressures are displayed in Figure 7 and Figure 8. Deviatoric stress q-$\varepsilon_{1}$ and columetric strain ε_v_-$\varepsilon_{1}$ relationships predicted by the state-dependent elasto-plastic model broadly match the experimental data [59], where a significant increase in perimeter pressure led to a corresponding increase in the stiffness and strength of cemented sand notably, and the peak intensity of the curve increases with increasing perimeter pressure. Because of the presence of bond strength and an increase in the critical stress ratio, the peak strength of hydrate-bearing cemented sand is higher than that of substrate sand. It is theorized that there is a small discrepancy in the model calculation when the perimeter pressure is lower since the predicted curve peak strength is smaller than the test curve. The stress-strain curve of the cemented sand develops towards strain hardening as the perimeter pressure increases, as evidenced by the continual saturation of hydrate and the disappearance of the softening tendency with the gradient of the perimeter pressure. The inclusion of the damage component in the model, which accounts for both the damage and the breakdown of hydrate, is likely the reason why the peak intensity of the simulated curve is marginally lower than that of the experimental curve.

**Figure 9:**
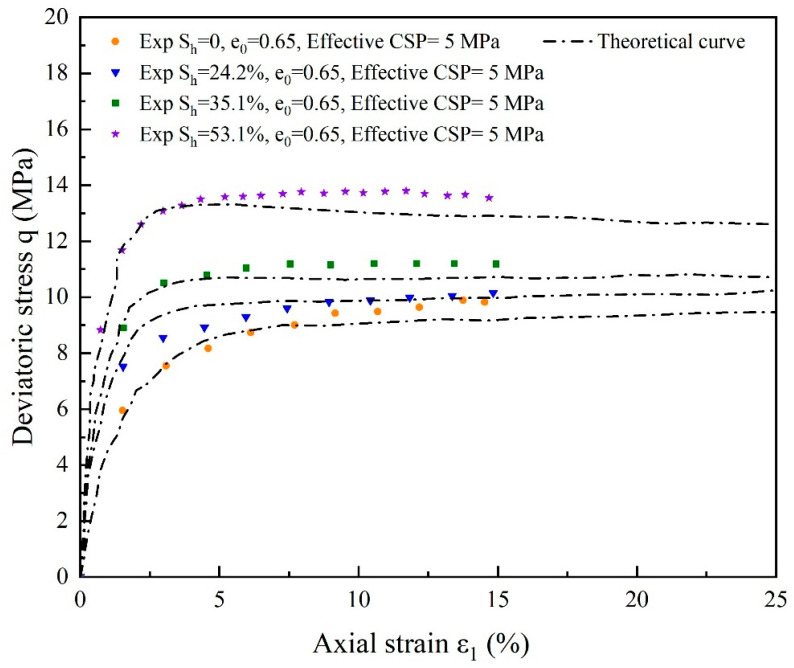
Comparing the theoretical and experimental deviatoric stress curves for cemented sands with hydrate saturation levels of 24.2%, 35.1%, and 53.1% and substrate sands with the same initial porosity (source of experimental data: Hyodo et al. [59]).

**Figure 10:**
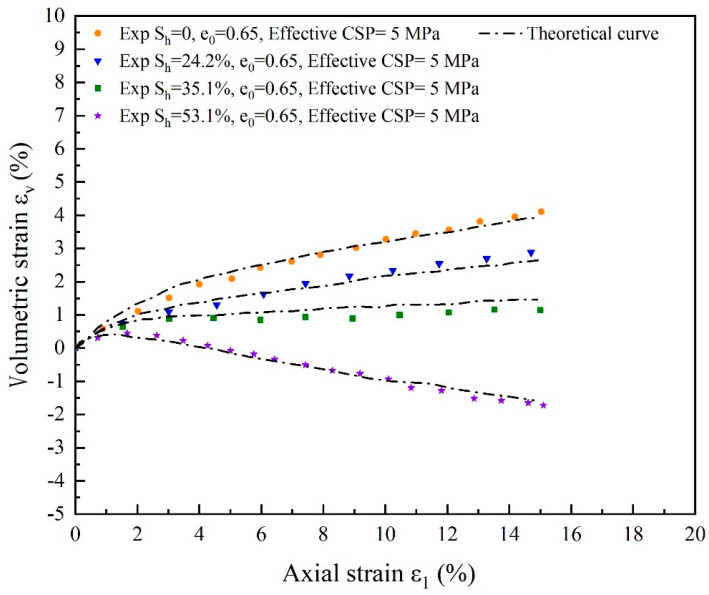
Comparing the theoretical and experimental volumetric strain for cemented sands with hydrate saturation levels of 24.2%, 35.1%, and 53.1% and substrate sands with the same initial porosity (source of experimental data: Hyodo et al. [59]).

For substrate sand with the same initial porosity and hydrate-bearing cemented sand with 24.2%, 35.1%, and 53.1% hydrate saturation, the deviatoric stress and volumetric strain simulation results are compared in Figure 9 and Figure 10. The pattern of strain softening with increasing hydrate-bearing cemented sand stiffness and maximum strength with increasing hydrate saturation can be adequately captured by the model since it accounts for both cementation factors and damage factors. As the effective restricting pressure decreases, the dilatancy becomes more noticeable. The curve’s peak strength appears at a very similar axial strain of about 2.3%. The maximum strength of the curve occurs earlier at hydrate saturations of 35.1% and 53.1% than at hydrate saturations of 24.2% and 0 at an axial strain of 2.1%, and the curve changes more steeply at the inflection point. This suggests that when the hydrate content rises, the hydrate and the yield deformation of soil break down sooner and more quickly. The soil can withstand more damage than gas hydrates, and its strength can be strengthened by increasing saturation.

**Figure 11:**
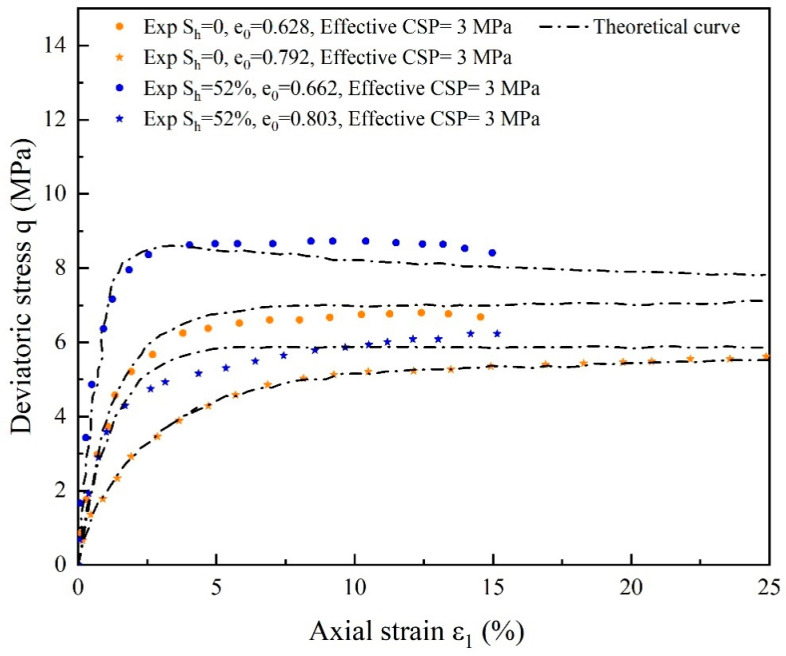
Comparison of experimental and theoretical curves of deviatoric stress for substrate sands with varying initial void ratio and cemented sands with 52% hydrate saturation (source of experimental data: Hyodo et al. [59]).

**Figure 12:**
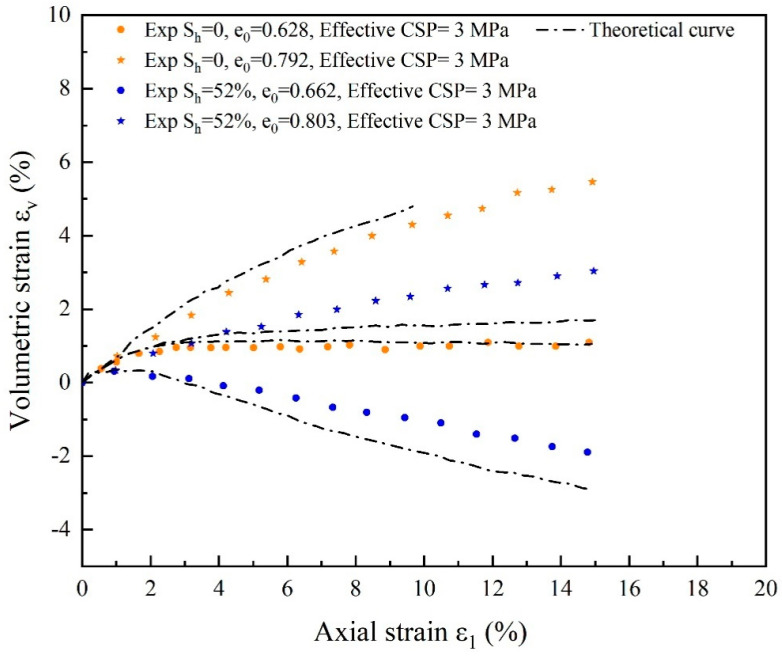
Comparison of experimental and theoretical curves of volumetric strain for substrate sands with varying initial void ratio and cemented sands with 52% hydrate saturation (source of experimental data: Hyodo et al. [59]).

The general trend of deformation of substrate sand and hydrate-bearing cemented sand with 52% saturation for various initial porosities is shown in Figure 11 and Figure 12. In Figure 8, as the hydrate saturation increases, the deviation of the theoretical simulation curves from the experimental curves of the volumetric strain $\varepsilon_{v}$- axial strain $\varepsilon_{1}$ relationship becomes more pronounced. This behavior could be explained by the fact that strain softening weakens and the strength of hydrate-bearing cemented sand decreases dramatically with increasing hydrate saturation. This suggests that while dense specimens show slight volume expansion behavior and strain softening, loose specimens exhibit compression behavior and strain hardening. There is a significant difference between the experimental data for a restricting pressure of 3 MPa and the predicted deviatoric stress *q* and volumetric strain *ε*_v_, in contrast to the curves for an effective confinement pressure of 5 MPa shown by Figure 9 and Figure 10. The model introduces cementation factor, and the stress-strain relationship behaves more clearly with increasing hydrate saturation relative to the conversion of the experimental data from strain hardening to softening. It is demonstrated that the model for hydrate-bearing cemented sand with cementation damage, which considers the state dependency, is valid and can accurately simulate both the mechanical characteristics of cemented sand damage and the weakening of cementation.

## 5. Conclusions

(1) A nonlinear state-dependent elasto-plastic constitutive model based on cementation and damage in hydrate-bearing cemented sand is constructed for the phenomena of natural gas hydrate cementation weakening and soil damage. This is achieved by introducing a quantitative description of the hydrate’s cementation and decomposition mechanism, taking into account the damage factor *D*_s_ and the cementation damage strength factor *P*_h_. The model captures the deviatoric stress *q*, volumetric strain *ε*_v_, and axial stress of hydrate-bearing cemented sands with acceptable accuracy at different hydrate saturation, effective confining pressure, and void ratio levels, when compared to data from drainage triaxial tests by Hyodo et al. [59] and other scholars, suggesting that the model is valid in representing the intricate mechanical behavior of compromised cementation and soil particles degradation in cemented sand.
(2) The state parameters represent the potential strength of cemented soil, and further introduce the state-dependent dilatancy equations. Compared to the previous model, a set of model parameters can be used to reproduce the mechanical properties of hydrate-bearing cemented sand with varying effective perimeter pressures, hydrate saturations, and void ratios. The strength of hydrate-bearing cemented sand against deviatoric stress increased with increasing hydrate saturation and surrounding pressure, and the peak strength was higher than that of substrate sand. The dilatancy phenomenon becomes more pronounced with decreasing effective confining pressure. Additionally, this agrees with experimental results of Hyodo et al. [59]. For different porosities, the loose specimens show strain hardening and compression behavior, and the dense specimens show slight strain softening and volume expansion behavior, with minor deviations from the model calculations at lower peripheral pressures.
(3) The simulated curves have slightly lower peak intensities compared to the experimental curves due to the inclusion of damage factors in the model that consider soil deterioration and gas hydrate decomposition. As the saturation of the gas hydrate increases, both the soil yield and the gas hydrate become more susceptible to distortion and breakdown. Unlike previous hydrate models, the damage index s controls the magnitude of the peak intensity of the stress-strain curve by measuring the rate of damage to the sand. The gas hydrate decomposition rate can be modified based on the impact of different external elements, which indicates the mechanical damage characteristics of cemented sand containing cemented hydrate under intricate circumstances.
(4) This study exclusively focuses on the cementing impact of hydrate-bearing cemented sand, disregarding other mechanical consequences of hydrate, such as the reduction in sediment porosity. Consequently, the model utilized in this study requires improvement. The next step will consist of examining the legal consequences of hydrate cementation damage and developing a constitutive model that incorporates hydrate compaction.

## Figures and Tables

**Table 1:** Model parameters of the substrate sand.

Symbol	Physical Meaning of Parameters	Value (Dimensionless)
μ	Poisson’s ratio	0.05
*G*_p_	The shear modulus factor	300
$\gamma_{c}$	Model parameter, the slope of lines of critical state	0.127
M	Critical stress ratio for substrate sand	1.16
$e_{\varpi }$	Model parameter for void ratio of substrate sand	1.119
*n*_0_	Model parameter for dilatancy	1.62
b	Model parameter for dilatancy	1.31
*l*_1_	The plastic modulus parameter	1.96
*l*_2_	The plastic modulus parameter	2.23
j	Model hardening rule parameter	0.78

**Table 2:** Extra model parameters for hydrate-bearing cemented sand.

Symbol	Physical Meaning of Parameters	Value (Dimensionless)
τ	An elastic modulus parameter denoting the gradient of the simulation line in Figure 1	215.7
ς	The critical state model parameters	0.58
κ	The critical state model parameters	1.99
χ	The critical state model parameters	0.36
δ	The critical state model parameters	1.38
g	Bonding parameter	1.59
c	Bonding parameter	0.67
*v*_c_	Debonding model parameter density on the plastic modulus	7.89
s	Damage factor	−0.10

## Data Availability

Data will be made available on request.

## Nomenclature

Symbol	Explanatory note
$\sigma_{1}^{*}$	The major primary stress
$\sigma_{3}^{*}$	The minor primary stress
$d\varepsilon_{1}$	The major principal strain increment
$d\varepsilon_{3}$	The minor principal strain increment
$d\varepsilon_{v}^{e}$	The elastic volumetric strain increment
$d\varepsilon_{v}^{p}$	The plastic volumetric strain increment
$d\varepsilon_{d}^{e}$	The elastic shear strain increment
$d\varepsilon_{d}^{p}$	The plastic shear strain increment
*D*_s_	The damage variable
$S_{h}$	The hydrate saturation
*G*	The elastic shear parameter
*K*	The elastic bulk parameter
*μ*	Poisson’s ratio
*P*_0_	The atmospheric pressure
*G*_p_	The shear stiffness parameter
$\eta^{*}$	The stress ratio at yielding
*K*_p_	The parameter of plastic hardening
*M*	The critical stress ratio
$e_{\varpi }$	The void ratio of sand
*D*	The dilatancy equation

## References

[1] Gajanayake S.M., Gamage R.P., Li X.S., Huppert H. (2023). Natural gas hydrates–Insights into a paradigm-shifting energy resource. Energy Rev..

[2] Ruan X., Li X.S., Xu C.G. (2021). A review of numerical research on gas production from natural gas hydrates in China. J. Nat. Gas Sci. Eng..

[3] Boswell R., Collett T.S. (2011). Current perspectives on gas hydrate resources. Energy Environ. Sci..

[4] Milkov A.V. (2004). Global estimates of hydrate-bound gas in marine sediments: How much is really out there?. Earth-Sci. Rev..

[5] Wang Y., Cui X., Che Y., Li P., Jiang Y., Peng X. (2023). Identification and Analysis of Unstable Slope and Seasonal Frozen Soil Area along the Litang Section of the Sichuan–Tibet Railway, China. Remote Sens..

[6] Zhu Y.H., Pang S.J., Xiao R., Zhang S., Lu Z.Q. (2021). Natural gas hydrates in the Qinghai-Tibet Plateau: Characteristics, formation, and evolution. China Geol..

[7] Gajanan K., Ranjith P.G., Yang S.Q., Xu T. (2024). Advances in research and developments on natural gas hydrate extraction with gas exchange. Renew. Sustain. Energy Rev..

[8] Dong H., Sun J., Arif M., Liu X., Golsanami N., Yan W., Zhang Y. (2022). A Method for Well Logging Identification and Evaluation of Low-Resistivity Gas Hydrate Layers. Pure Appl. Geophys..

[9] Both A.K., Gao Y., Zeng X.C., Cheung C.L. (2021). Gas hydrates in confined space of nanoporous materials: New frontier in gas storage technology. Nanoscale.

[10] Chen Y., Tong H., Chen Q., Du X., Wang S., Pan Y. (2023). Chemical Corrosion-Water-Confining Pressure Coupling Damage Constitutive Model of Rock Based on the SMP Strength Criterion. Materials.

[11] Liu G., Chen Y., Du X., Xiao P., Liao S., Azzam R. (2021). Investigation of Microcrack Propagation and Energy Evolution in Brittle Rocks Based on the Voronoi Model. Materials.

[12] Liu G., Chen Y., Du X., Wang S., Fernández-Steeger T.M. (2022). Evolutionary Analysis of Heterogeneous Granite Microcracks Based on Digital Image Processing in Grain-Block Model. Materials.

[13] Tong H., Chen Y., Chen Q., Du X., Xiao P., Wang S. (2023). A true triaxial creep constitutive model of rock considering the coupled thermo-mechanical damage. Energy.

[14] Makogon Y.F. (2010). Natural gas hydrates—A promising source of energy. J. Nat. Gas Sci. Eng..

[15] You K., Flemings P.B., Malinverno A., Collett T.S., Darnell K. (2019). Mechanisms of methane hydrate formation in geological systems. Rev. Geophys..

[16] Ren J., Liu X., Niu M., Yin Z. (2022). Effect of sodium montmorillonite clay on the kinetics of CH4 hydrate—Implication for energy recovery. Chem. Eng. J..

[17] Yoneda J., Jin Y., Katagiri J., Tenma N. (2016). Strengthening mechanism of cemented hydrate-bearing sand at microscales. Geophys. Res. Lett..

[18] Waite W.F., Winters W.J., Mason D.H. (2004). Methane hydrate formation in partially water-saturated Ottawa sand. Am. Mineral..

[19] Silvestri V. (1981). Behavior of an Oviangerconsolidated Sensitive Clay in Drained—Triaxial Tests. Lab. Shear. Strength Soil.

[20] Abbasi G.R., Arif M., Isah A., Ali M., Mahmoud M., Hoteit H., Iglauer S. (2022). Gas hydrate characterization in sediments via x-ray microcomputed tomography. Earth-Sci. Rev..

[21] Brown H.E., Holbrook W.S., Hornbach M.J., Nealon J. (2006). Slide structure and role of gas hydrate at the northern boundary of the Storegga Slide, offshore Norway. Mar. Geol..

[22] Miyazaki K., Tenma N., Aoki K., Yamaguchi T. (2012). A Nonlinear Elastic Model for Triaxial Compressive Properties of Artificial Methane-Hydrate-Bearing Sediment Samples. Energies.

[23] Ng C.W.W., Baghbanrezvan S., Kadlicek T., Zhou C. (2020). A state-dependent constitutive model for methane hydrate-bearing sediments inside the stability region. Géotechnique.

[24] Shen J., Chiu C.F., Ng C.W.W., Lei G.H., Xu J. (2016). A state-dependent critical state model for methane hydrate-bearing sand. Comput. Geotech..

[25] Sultan N., Garziglia S. Geomechanical constitutive modelling of gas-hydrate-bearing sediments. Proceedings of the ICGH 2011.

[26] Uchida S., Soga K., Yamamoto K. (2012). Critical state soil constitutive model for methane hydrate soil. J. Geophys. Res..

[27] Hyodo M., Li Y., Yoneda J., Nakata Y., Yoshimoto N., Nishimura A. (2014). Effects of dissociation on the shear strength and deformation behavior of methane hydrate-bearing sediments. Mar. Petrol. Geol..

[28] Zhang X.H., Luo D.S., Lu X.B., Liu L.L., Liu C.L. (2018). Mechanical properties of gas hydrate-bearing sediments during hydrate dissociation. Acta Mech. Sin..

[29] Li Y., Wu P., Sun X., Liu W., Song Y. (2021). Mechanical behaviors of hydrate-bearing sediment with different cementation spatial distributions at microscales. iScience.

[30] Wang L.Z., Li L.L. (2006). Valuation of Poisson’s ratio in nonlinear elastic modeling of structural soft soils. J. Water. Res..

[31] Yoshimoto M., Kimoto S. (2022). Undrained creep behavior of CO_2_ hydrate-bearing sand and its constitutive modeling considering the cementing effect of hydrates. Soils. Found.

[32] Iwai H., Kawasaki T., Zhang F. (2022). Constitutive model for gas hydrate-bearing soils considering different types of hydrate morphology and prediction of strength-band. Soils. Found.

[33] Bhattacharjee G., Veluswamy H.P., Kumar A., Linga P. (2021). Asheesh Kumar a c, Praveen Linga a. Stability analysis of methane hydrates for gas storage application. Chem. Eng. J..

[34] Fu Z., Chen S., Zhong Q., Ji E. (2022). A damage hypoplasticity constitutive model for cemented sand and gravel materials. Acta Geotech..

[35] Almenningen S., Gauteplass J., Fotland P., Aastveit G.L., Barth T., Ersland G. (2018). Visualization of hydrate formation during CO2 storage in water-saturated sandstone. Int. J. Greenh. Gas. Control.

[36] Yan R.T., Liang W.Y., Wei C.F., Wu E.L. (2017). Ontological modeling of hydrate-bearing sediments taking into account the influence of storage mode. Geotech. Mechan..

[37] Yin S., Hou Y., Chen X., Zhang M., Du H., Gao C. (2022). Mechanical behavior, failure pattern and damage evolution of fiber-reinforced cemented sulfur tailings backfill under uniaxial loading. Constr. Build. Mater..

[38] De La Fuente M., Vaunat J., Marín-Moreno H. (2020). A densification mechanism to model the mechanical effect of methane hydrates in sandy sediments. Int. J. Numer. Anal. Met..

[39] Zhu E.Y., Li X.Q. (2018). Uniform hardening model for cemented structural soils. Geotechnics.

[40] Ji F.L., Lu Q.F., Li M.D. (2006). Structural Modeling of Lightweight Mixed Soil with Dredged Silt EPS Particles. J. Shenzhen Univ..

[41] Nguyen L.D., Fatahi B., Khabbaz H. (2014). A constitutive model for cemented clays capturing cementation degradation. Int. J. Plast..

[42] Li X.S., Dafalias Y.F., Wang Z.L. (1999). State-dependant dilatancy in critical-state constitutive modelling of sand. Can. Geotech. J..

[43] Li X.S., Dafalias Y.F. (2000). Dilatancy for cohesionless soils. Géotechnique.

[44] Suebsuk J., Horpibulsuk S., Liu M.D. (2011). A critical state model for overconsolidated structured clays. Comput. Geotech..

[45] Drucker D.C., Gibson R.E., Henkel D.J. (1957). Soil Mechanics and Work-Hardening Theories of Plasticity. Tran. Am. Soc. Civil. Eng..

[46] Roscoe K.H., Burland J.B. (1968). On the generalized stress-strain behaviour of wet clay. Engineering Plasticity.

[47] Roscoe K.H., Schofield A.N., Thurairajah A. (1963). Yielding of Clays in States Wetter than Critical. Géotechnique.

[48] DiMaggio F.L., Sandler I.S. (1997). Material Model for Granular Soils. J. Eng. Mech. Div..

[49] Sandler I.S., Baladi G.Y., DiMaggio F.L. (1976). Generalized Cap Model for Geological Materials. J. Geotech. Eng. Div..

[50] Huang T.K., Chen W.F. (1990). Simple Procedure for Determining Cap-Plasticity-Model Parameters. J. Geotech. Eng..

[51] Zhang Y.B., Wen X., Jin J.K., Zhao C.H. (2023). An elasto-plastic constitutive model for deep-sea energy soils considering the effects of cementation and damage. Ind. Technol. Vocat. Educ..

[52] Kim J., Dai S., Jang J., Waite W.F., Collett T.S., Kumar P. (2019). Compressibility and particle crushing of Krishna-Godavari Basin sediments from offshore India: Implications for gas production from deep-water gas hydrate deposits. Mar. Petrol. Geol..

[53] Consoli N.C., Cruz R.C., Da Fonseca A.V., Coop M.R. (2012). Influence of Cement-Voids Ratio on Stress-Dilatancy Behavior of Artificially Cemented Sand. J. Geotech. Geoenviron. Eng..

[54] Xiao Y., Liu H., Chen Y., Jiang J., Zhang W. (2014). Testing and modeling of the state-dependent behaviors of rockfill material. Comput. Geotech..

[55] Clayton C.R.I., Priest J.A., Rees E.V.l. (2010). The effects of hydrate cement on the stiffness of some sands. Géotechnique.

[56] Manzari M.T., Dafalias Y.F. (1997). A critical state two-surface plasticity model for sands. Géotechnique.

[57] Dafalias Y.F. (1986). An anisotropic critical state soil plasticity model. Mech. Res. Commun..

[58] Hyodo M., Li Y., Yoneda J., Nakata Y., Yoshimoto N., Nishimura A. (2013). Mechanical behavior of gas-saturated methane hydrate-bearing sediments. J. Geophys. Res. Solid Earth.

[59] Hyodo M., Yoneda J., Yoshimoto N., Nakata Y. (2013). Mechanical and dissociation properties of methane hydrate-bearing sand in deep seabed. Soils Found..

[60] Hyodo M., Yamamoto Y., Sugiyama M. (1994). Undrained cyclic shear behaviour of normally consolidated clay subjected to initial static shear stress. Soils Found..

[61] Masui A., Haneda H., Ogata Y., Aoki K. Effects of Methane Hydrate Formation on Shear Strength of Synthetic Methane Hydrate Sediments. Proceedings of the ISOPE International Ocean and Polar Engineering Conference.

[62] Miyazaki K., Masui A., Sakamoto Y., Aoki K., Tenma N., Yamaguchi T. (2011). Triaxial compressive properties of artificial methane-hydrate-bearing sediment. J. Geophys. Res. Solid Earth..

[63] Been K., Jefferies M.G. (1986). Discussion: A state parameter for sands. Géotechnique.

[64] Chiu C.F., Ng C.W.W. (2003). A state-dependent elasto-plastic model for saturated and unsaturated soils. Géotechnique.

